# The potential involvement of inhaled iron (Fe) in the neurotoxic effects of ultrafine particulate matter air pollution exposure on brain development in mice

**DOI:** 10.1186/s12989-022-00496-5

**Published:** 2022-08-09

**Authors:** Marissa Sobolewski, Katherine Conrad, Elena Marvin, Matthew Eckard, Calla M. Goeke, Alyssa K. Merrill, Kevin Welle, Brian P. Jackson, Robert Gelein, David Chalupa, Günter Oberdörster, Deborah A. Cory-Slechta

**Affiliations:** 1 Department of Environmental Medicine, University of Rochester Medical Center, Rochester, NY 14642 USA; 2 Department of Psychology, Radford University, Radford, VA 24142 USA; 3 Proteomics Core, University of Rochester Medical Center, Rochester, NY 14642 USA; 4 Department of Earth Sciences, Dartmouth College, Hanover, NH 03755 USA

**Keywords:** Ultrafine particulate matter, Iron, Sulfur dioxide, Neurodevelopmental disorders, Brain, Metal dyshomeostasis

## Abstract

**Background:**

Air pollution has been associated with neurodevelopmental disorders in epidemiological studies. In our studies in mice, developmental exposures to ambient ultrafine particulate (UFP) matter either postnatally or gestationally results in neurotoxic consequences that include brain metal dyshomeostasis, including significant increases in brain Fe. Since Fe is redox active and neurotoxic to brain in excess, this study examined the extent to which postnatal Fe inhalation exposure, might contribute to the observed neurotoxicity of UFPs. Mice were exposed to 1 µg/m^3^ Fe oxide nanoparticles alone, or in conjunction with sulfur dioxide (Fe (1 µg/m^3^) + SO_2_ (SO_2_ at 1.31 mg/m^3^, 500 ppb) from postnatal days 4–7 and 10–13 for 4 h/day.

**Results:**

Overarching results included the observations that Fe + SO_2_ produced greater neurotoxicity than did Fe alone, that females appeared to show greater vulnerability to these exposures than did males, and that profiles of effects differed by sex. Consistent with metal dyshomeostasis, both Fe only and Fe + SO_2_ exposures altered correlations of Fe and of sulfur (S) with other metals in a sex and tissue-specific manner. Specifically, altered metal levels in lung, but particularly in frontal cortex were found, with reductions produced by Fe in females, but increases produced by Fe + SO_2_ in males. At PND14, marked changes in brain frontal cortex and striatal neurotransmitter systems were observed, particularly in response to combined Fe + SO2 as compared to Fe only, in glutamatergic and dopaminergic functions that were of opposite directions by sex. Changes in markers of trans-sulfuration in frontal cortex likewise differed in females as compared to males. Residual neurotransmitter changes were limited at PND60. Increases in serum glutathione and Il-1a were female-specific effects of combined Fe + SO2.

**Conclusions:**

Collectively, these findings suggest a role for the Fe contamination in air pollution in the observed neurotoxicity of ambient UFPs and that such involvement may be different by chemical mixture. Translation of such results to humans requires verification, and, if found, would suggest a need for regulation of Fe in air for public health protection.

**Supplementary Information:**

The online version contains supplementary material available at 10.1186/s12989-022-00496-5.

## Background

Numerous epidemiological studies now associate exposures to air pollution (AP) with various neurodevelopmental disorders, including autism spectrum disorder (ASD), schizophrenia (SCZ) and attention deficit hyperactivity disorder (ADHD), all male-biased disorders. Experimental animal models increasingly provide biological plausibility for these associations [1–9]. In our studies in mice that underwent early postnatal exposures (equivalent to human third trimester brain development [10, 11]) to concentrated ambient ultrafine particles (UFPs), numerous and persistent male-specific or male-biased neuropathological changes and alterations in behavioral functions were observed, including ventriculomegaly, elevated brain glutamate levels, reductions in size and myelination of corpus callosum (the largest white matter tract in brain), increases in cytokine levels, and behavioral deficits including impulsive-like behavior [12–16]. When such exposures were carried out gestationally [17–19], ventriculomegaly was still observed, although in this period of exposure it was seen in both sexes, as was, in direct contrast to postnatal exposures, an increase in the size and myelination of corpus callosum, with collective effects that were more female-biased. These pathological features are consistent with numerous neurodevelopmental disorders [20–29] and thereby underscore the need to understand the components of air pollution that result in this neurotoxicity and how the impacts of these different components may differ by sex.

Analyses of filters from exposure chambers in our studies revealed the presence of multiple metals and trace elements in the AP exposures that could lead to brain metal dyshomeostasis [30]. Iron (Fe) is the metal of greatest abundance in the atmosphere [31] and thus is often a dominant component of the trace element levels of particulate matter, with concentrations clearly differing by geographical and industrial source. Indeed, a study from Korea reported that Fe dominated the PM_2.5_ mass in subway stations at a concentration of > 100 µg/m^3^ [32] while New York city subways were found to range from 141 to 329 µg/m^3^ [32]. A study from China reported median Fe concentrations in outdoor air samples in Bejing at concentrations of 0.614 µg/m^3^ in August and 0.378 µg/m^3^ in December [33]. In a comparative study, Fe concentrations in Lodz Poland averaged 0.677 µg/m^3^, and corresponding values for Milan averaged 0.353 µg/m^3^ [34] whereas values from Karachi Pakistan averaged 3.175 µg/m^3^ [35]. Data from the U.S. Environmental Protection Agency Speciate Database cited Fe emissions of 103,000 tons/year and 1210 µg/m^3^/day [36].

Correspondingly, ICP-MS analyses of brain sections from male mice exposed postnatally to concentrated ambient ultrafine particles revealed brain metal dyshomeostasis, characterized by marked elevations of metals and trace elements in brain, including Fe and sulfur (S), but that also included increases in calcium (Ca), copper (Cu) and aluminum (Al) [30]. Increases in brain Fe were also found in response to gestational exposures, particularly in female offspring [19]. While both Fe and S are requisite to brain development [37–42], they are also detrimental in excess [43–49]; Fe in air pollution has been shown to be redox active and capable of inducing oxidative stress [50]. Mechanisms to control Fe uptake in brain are not operative during early periods of brain development [51], leaving fetal brain susceptible to such exposures.

Metal dyshomeostasis has been implicated in neurodevelopmental disorders including autism, schizophrenia and attention deficit disorder [52–57]. While in the case of neurodevelopmental disorders such reports have largely been based on serum or hair analyses, studies have reported excess Ca levels in post-mortem neocortical tissue from individuals that had been diagnosed with ASD [58], and serum metal changes have been related to markers of inflammation in autism as well [59]. Understanding of changes in brain metals specifically and their relationships to neurodevelopmental disorders is not clear, particularly as directions of changes in metals in serum do not necessarily correspond to alterations in levels of those metals in brain [60–64].

Based on these findings, the current study sought to determine the extent to which the Fe component, via Fe oxide nanoparticle inhalation, serves as a basis for the neurotoxicity produced by postnatal inhaled ambient ultrafine particle exposures. In ambient air, Fe concentrations are correlated with sulfate content, based on sulfate’s ability to mobilize Fe from its oxide form [65]. Further, an early study [66] reported that sulfur dioxide (SO_2_) increased the uptake of Fe into the central nervous system (CNS) and altered its distribution among different cell types. Consequently, in other cohorts of mice, Fe oxide nanoparticle exposures were carried out concurrently with SO_2_ exposure (Fe + SO_2_) and assessments of metal dyshomeostasis, trans-sulfuration pathways, serum cytokines and brain neurotransmitter levels were compared to effects previously observed in response to ambient UFP exposures from our prior studies [12, 14, 15, 67].

## Methods and materials

### Animals

C57BL6/J mice were kept, bred and exposed as previously described [12, 14, 68–76]. Briefly, mice were bred monogamously with sires removed following sperm plug identification. Pups were housed solely with the dam and were weaned on postnatal day (PND) 21. Mice were housed in standard mouse caging with 1/8″ high performance bedding (BioFresh, WA, USA), under a 12 h light-dark cycle, maintained at 22 ± 2 °C, and fed standard rodent chow at the University of Rochester Medical Center. Following weaning, offspring were pair housed by sex and treatment group for the duration of the study. All mice were used and treated via protocols approved by the University of Rochester Medical Center Institutional Animal Care and Use Committee and Committee on Animal Resources (approval # 102,208 / 2010-046E), and in accordance with NIH guidelines. Mice were euthanized at either PND 14, 30, 60 or 90 and tissue harvested for various analyses.

### Fe and Fe + SO_2_ exposures

Mice were exposed either to iron (Fe) and and or Fe and sulfur (S); specifically, to Fe oxide (Fe) nanoparticles alone or with sulfur dioxide (SO_2_) gas from postnatal days 4–7 and 10–13 for 4 h. The exposures were to Fe vs Fe + SO_2_ were carried out at separate times, each with its corresponding filtered air control group. Mice were exposed in exposure cages via whole body inhalation. For this study, the intended SO_2_ concentration was 1.31 mg/m^3^, and the intended Fe concentration was 1.0 µg/m^3^. The Fe concentration was chosen to be within the range of values cited for outdoor Fe levels as cited above [33–35]. The SO_2_ concentration was based on the U. S. Environmental Protection Agency secondary standard for SO_2_. Fe-oxide UFP particles were generated by electric spark discharge between two 99.99999% pure iron rods (3N5 Purity, ESPI Metals, Ashland, OR, USA) using a GFG-1000 Palas generator (Palas GmbH, Karlshrue, Germany), and fed into a compartmentalized whole-body mouse exposure chamber, while HEPA-filtered air was delivered to the control chamber, as in prior studies in our inhalation facility [77]. Passing the airborne particles through a deionizer (Isotope Po-210, model P-2031, NRD, Grand Island, NY, USA) was used to bring particle charge to Boltzmann equilibrium. Particle number concentration was adjusted by altering electric spark discharge frequency. Aerosol number concentration and particle size were monitored in real-time using a Condensation Particle Counter (CPC, model 3022, TSI Inc, St Paul, MN, USA) and Scanning Mobility Analyzer (SMPS, model 3934 TSI Inc, St Paul, MN, USA) respectively. The Fe-oxide particles were generated by adding a low flow of oxygen (~ 50 mL/min) into the argon flow (~ 5 L/min) entering the spark discharge chamber. The oxygen concentration of 21% in the exposure chamber was verified by an O_2_ sensor (MAXO2 -250E, Maxtec, Salt Lake City, UT, USA). This procedure produced particle sizes exclusively in the ultrafine size range with a count median diameter (CMD) of approximately 12–14 nm. Mass concentrations were determined by ICP-OES analysis of Fe on nitrocellulose membrane filters (0.8 micron, AAWP02500, Millipore Ltd., Tullagreen, Cork, IRL) collected daily (5 L/min for 60 min., 300L total volume) from the filtered air and ultrafine Fe-oxide particle exposure chambers. For the concurrent SO_2_ exposures, SO_2_, compressed in gas cylinders (EPA Protocol Standard, 50 ppm, Airgas East, Radnor, PA, USA), was diluted with filtered air and then bled into (200 ml/min) the Fe-oxide containing conduit to achieve final desired concentrations for Fe + SO_2_ exposures. This Fe-oxide/SO_2_ mixture was fed into the whole-body exposure chamber at 25–30 L per minute. SO_2_ concentrations were continuously monitored and recorded with an SO_2_ gas monitor (model 43C, Thermo Environmental Instruments Inc., Franklin, MA, USA). Intended Fe and Fe + SO_2_ concentrations were chosen to be consistent with the lower reported concentrations as cited above.

### Metal analyses

Pre-weighed tissue samples were acid digested in 1.5 ml Eppendorf tubes using a Fisher hot-block. A sample-dependent range of 200–500 µl of 9:1 HNO3:HCl (Optima grade, Fisher Scientific) was added to the tubes, which were then heated at 80 C for 1 h. Olfactory bulbs (1–9 mg) were digested with 200 µl acid mix and diluted to a final volume of 2 ml with deionized water and the final dilution weight recorded. Cortex (48–103 mg) and lung (15–96 mg) were digested with 500 µl acid mix and diluted to a final volume of 5 ml with deionized water and the final dilution weight recorded. Six blanks and 6 reference material samples (NIST 2976 Mussel Tissue, 6–18 mg) were included in the digest and diluted to 5 ml.

Digested samples were analyzed by ICP-MS (Agilent 8900, Wilmington, DE) in helium and oxygen modes. Nist-traceable primary standards were used to construct a multi-element calibration curve, second source standards were used for a calibration check repeated after each calibration and every 10 samples. USGS water proficiency samples (P76, T-245) were used as a further calibration check and repeated three further times during the analysis. Five analytical duplicates and five analytical spikes were also performed and data reported in ug/g or ng/g depending upon the metal. Quality control data is summarized in Additional file 1: Table 1.

### Bronchoalveolar lavage (BAL) procedure and analysis

Lungs were excised with the trachea, then lavaged 10 times with warmed sterile 0.9% saline (10 × 1 mL). For this purpose, the first two lavage washes were reserved separately from the rest to prevent cell dilution. BAL fluid was centrifuged at 400 × g for 10 min to separate out the cell fraction. Supernatants from lavages 1–2 were used for measurements of total protein (microBCA, Thermo-Fisher Scientific) and lactate dehydrogenase (LDH) activity (Sigma Aldrich).

### Serum glutathione and IL-1a

Serum glutathione was measured using the Glutathione Colorimetric Detection Kit (Arbor Assays, Cat. K006-H1). The kit was run, as described, and each sample was run twice to measure both oxidized and total glutathione. Samples were run in duplicate and counter-balanced across the plate based on sex and treatment group.

Serum cytokines (IL-1a, IL-1b, IL-2, IL-6, IL10, IFN-g and TNF-a) were measured using Mouse Cytokine Grpl 7-plex luminex Kit (Bio-Rad, Cat. Y60000017G) as described in Bio-Plex Pro Assays for use with Mouse and Rat Cytokine Assays, instruction manual #10,014,905. Limits of detection for the Fe + SO_2_ samples were 0.508, 1.99, 1.397. 0.411, 13.197, 3.331 and 230.587 pg/ml, and for the Fe samples, were 2.248, 1.425, 1.335, 1.967, 4.694, 0.885 and 54.706, respectively. For both cohorts, samples were run in duplicate and counter-balanced across the plate based on sex and treatment group. Sample and standard duplicates calculated coefficients of variation were below 15%.

### Brain neurotransmitter and trans-sulfuration analyses

Striatal concentrations of various neurotransmitters were quantified by the University of Rochester Mass Spectrometry Core: DA, DOPAC, HVA, Tyrosine (Tyr), Glutamate (Glu), GABA, Glutamine (Gln), Kynurenic Acid (Kyn), 5-HT, 5-HIAA, and Tryptophan (Trp). Tissues were thawed, weighed, diluted in 75 µL of ice-cold acetonitrile (50%, v/v) and homogenized for 10 s via ultra-sonication (SLPe digital sonifier, Branson Ultrasonics Corp., Danbury, CT.). The homogenate was centrifuged at 10,000 g (4 °C) for 20 min. The resulting supernatant was collected and centrifuged at 10,000 g (4 °C) for 20 min, after which the new supernatant was collected and stored at -80 °C until analysis.

Stock solutions of DA, DOPAC, HVA, Glu, GABA, Glu, Kyn, 5-HT, 5-HTP, 5-HIAA, and Trp (Sigma Aldrich) were made at 5 mg/mL in ddH_2_O, with the exception of Tyr, which was made in 0.2 M HCl. A standard mixture was created in ddH_2_O, with analyte concentrations varying in accordance with prior range-finding studies, in order to account for region-specific variations in endogenous neurotransmitters. This stock solution was derivatized using 13C6 benzoyl chloride (BzCl, Sigma Aldrich) using a method adapted from Wong et al. [78], to create internal standards for each individual neurotransmitter. The derivatized internal standard mixture was aliquoted and frozen at − 80 °C for long term storage. Internal standard aliquots were thawed, then diluted in 50% acetonitrile with 1% sulfuric acid prior to being added to the samples. Prior to analysis, samples were derivatized following the same procedure. In brief, samples were centrifuged at 16,000 g for 5 min to remove debris, then 20 µL of resulting supernatant was placed in a clean LoBind tube (Eppendorf). Next, 10 µL of 100 mM sodium carbonate, 10 µL of 2% BzCl in acetonitrile, and 10 µL of the respective internal standard was added in sequence. 50 µL of ddH_2_O was then added to reduce the organic concentration prior to injection. Samples were centrifuged once more to pellet any remaining protein, and the supernatant was added to a clean autosampler vial.

LC-MS/MS analysis was carried out by a Dionex Ultimate 3000 UHPLC coupled to a Q Exactive Plus mass spectrometer (Thermo Fisher). Analytes were separated on a Waters Acquity HSS T3 column. The mobile phases were: A) 10 mM ammonium formate in 0.1% formic acid, and B) acetonitrile. The flow rate was set to 400 µL/min and the column oven was set at 27 °C. After 5 µL of each sample was injected, the analytes were separated using a 12 min multi-step gradient. The Q Exactive Plus was operated in positive mode, and a parallel reaction monitoring method (PRM) was used to detect derivatized molecules. Fragment ions were extracted with a 10 ppm mass error using the LC Quan node of the XCalibur software (Thermo Fisher). Endogenous analyte peak areas were compared to those of each internal standard to determine relative abundance. These values were then divided by wet weight of the sample and then calculated by air control to yield percent of control values.

### Statistical analysis

Levels of serum cytokines, striatal and frontal cortical neurotransmitters (area ratio/weight values), serum oxidized glutathione and metal levels in tissues were analyzed using t tests comparing the Fe or Fe + SO_2_ exposed mice to their corresponding control group. These analyses were carried out separately by sex, as our prior studies consistent reveal sex differences in response to air pollution [12, 14, 68]. To assess metal dyshomeostasis, multivariate correlation analysis was carried out across metals for lung, olfactory bulb and cortex. Body weights over the course of exposure to Fe + SO_2_ were analyzed using repeated measures analyses of variance based on Pearson’s correlation coefficients with time as a within-group factor and treatment as a between-group factor.

All data were analyzed using JMP Pro16; outliers were evaluated using a Grubb’s test (Prism Graph Pad); no more than one outlier was removed from any group (treatment/sex), and the mean value of the remaining values was substituted. A *p*-value of ≤ 0.05 was considered significant; marginal effects where reported reflect a *p* value of ≤ 0.10.

## Results

### Exposure characteristics

**Fig. 1:**
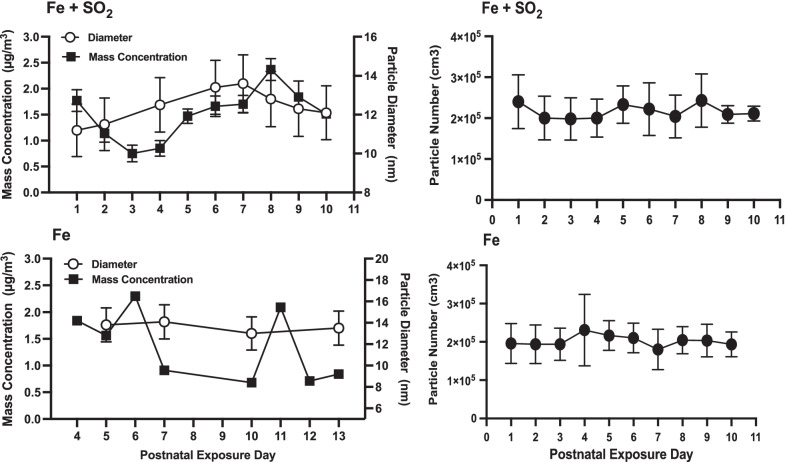
Left: Particle Diameter and Mass Concentration for Exposures: Particle diameter (nm) and mass concentration mean ± SD values (ug/m^3^) for the Fe + SO_2_ exposure (top row) and Fe only exposure (bottom row) across exposure days. Particle diameter ranged from 12.7 to 13.9 nm for Fe + SO_2_ and from 13.0 to 14.0  nm for Fe. Mass concentrations averaged from 0.75 to 2.37 ug/m^3^ for Fe + SO_2_ and 0.68 to 2.3 ug/m^3^ for Fe exposures. Right panel: Group mean ± SD particle number for Fe + SO_2_ exposure (top) and Fe only (bottom)

Particle diameter and mass as well as particle number for each of the two exposures are shown in Fig. 1. For Fe + SO_2_ (Fig. 1 top, Fe + SO_2_), Fe mass concentrations across days ranged from 0.75 to 2.37 µg/m^3^ over the exposure period, producing an average concentration of 1.51 µg/m^3^, given the known oxidation of Fe with relative contribution of oxygen to these particles and the intended target of 1.0 µg/m^3^ inhaled Fe. Count Median Diameter (CMD) particle size varied from 11.2 to 13.6 nm, with an average Geometrical Standard Deviation (GSD) of 1.4, which is within the ultrafine size range. The daily particle number concentration average ± standard deviation was 2.16E + 05 ± 0.17E + 05 part/cm^3^, a consistently high number concentration exposure of pure Fe particles, exceeding what would be seen in Fe ambient environmental exposure levels by number. Therefore, a large number to surface area ratio (µm^2^/cm^3^) consistently existed for these laboratory exposures, about 150 times that of a unit density sphere at a mass concentration of 1 ug/m^3^. For Fe only exposure (Fig. 1 bottom), Fe particle mass concentrations were 10-day average = 1.42 µg/m^3^, again given the known oxidation of Fe with relative contribution of oxygen to these particles with the intended 1 µg/m^3^ target on average for inhaled Fe, with variation across the 10 days (range = 0.68–2.44 µg/m^3^). The particle number concentrations were similar for Fe only exposures (10-day avg. ± st dev.: 2.03E + 05 ± 0.14E + 05 part/cm^3^). Particle diameter was consistently between 13–14 nm (mean = 13.6 nm, GSD 1.6) indicating a consistent ultrafine aerosol, as intended.

### Body weights

**Fig. 2:**
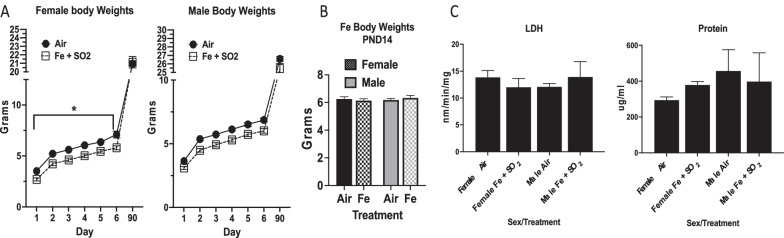
Panel **A**: Group mean ± S.E. body weight (gm) of female and male mice (n = 12/group) exposed to filtered air or Fe + SO_2_ over the course of exposure and in adulthood. * = Significant reductions ranging from 14 to 23% occurred in Fe + SO_2_ females, whereas differences in males were not statistically significant. Panel** B**: Group mean ± S.E. body weight (gm) at postnatal day 14 of female and male mice (n = 12/group) exposed to filtered air or Fe only. Panel **C**: Group mean ± S.E. changes in LDH (lactate dehydrogenase; nm/min/mg) and protein levels (ug/ml) in lung of male and female air versus Fe + SO_2_ treated mice showed no evident differences (n = 2/group)

Body weights of the Fe + SO_2_ group were over the course of the exposures as well as out to postnatal day 90  are shown in Fig. 2A. As can be seen, small but significant body weights reductions were seen in females exposed to Fe + SO_2_ relative to female air control over the course of the exposure, while similar but non-significant trends were observed in males (female: F(1,17) = 28.09, *p* < 0.0001; males: F(1,15) = 2.58, *p* = 0.13). However, these differences had disappeared by postnatal day 90, at which time there were no significant differences in either sex. In the Fe only exposed group (Fig. 2B), body weights were obtained at postnatal day 14 and did not differ in relation to exposure in either sex (females: 6.18 ± 0.11 versus 6.33 ± 0.108 gm for air-exposed vs. Fe-exposed; males: 6.26 ± 0.15 versus 6.13 ± 0.14 gm for air-exposed versus Fe-exposed).

### Lung markers

Bronchioalveolar lavage fluid was collected from Fe + SO_2_ exposed pups to ascertain markers of lung function, including lactate dehydrogenase (LDH) and total protein levels, and corresponding values are shown in Fig. 2C. These assessments did not reveal any evident  changes in response to Fe + SO_2_ in either sex.

### Metal levels and correlations of metals

**Fig. 3:**
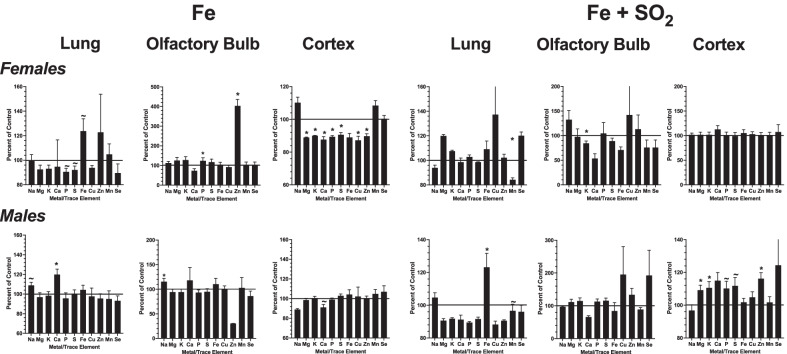
Group mean ± S.E. changes in concentrations of metals and trace elements plotted as a percent of filtered air control levels following Fe exposure (left 3 panels) or Fe + SO_2_ exposure (right 3 panels) for females (top row) and males (bottom row) in lung (first panel), olfactory bulb (middle panel) and frontal cortex (rightmost panels). * = significantly different from filtered air control; ~  = marginally different from filtered air control; n = 4/group

Levels of metals and trace elements were quantified (µg/gm or ng/g of tissue) in lung, olfactory bulb and frontal cortex, and values are presented as percent of filtered air control levels in **Fig. ****3**. After Fe only exposures, the most dramatic effects in females were found in frontal cortex, with reductions in levels of Mg, K, Ca, P, S, Cu and Zn (Mg: F(1,6) = 27.4, *p* = 0.002; K: F(1,6) = 14.18, *p* = 009; Ca: F(1,6) = 5.998, *p* = 0.0499; P: F(1,6) = 12.13, *p* = 0.013; S: F(1,6) = 6.38, *p* = 0.045; Cu: F(1,6) = 20.6, *p* = 0.004; Zn: F(1.6) = 11.3, *p* = 0.015), with a marked increase in Zn in olfactory bulb (F(1,6) = 82.86, *p* < 0.001), and marginal reductions of P and S in lung (P: F(1,6) = 5.87, *p* = 0.051; S (F91,6) = 3.79, *p* = 0.099). In males, the only significant changes in response to Fe were marginal increases in Na (F(1,6) = 5.81, *p* = 0.053) and Ca in lung (F(1,6) = 11.49, *p* = 0.015) and a marginal reduction in Ca in frontal cortex (F(1,6) = 4.13, *p* = 0.089).

Following Fe + SO_2_ exposures, females showed significant reductions in Mn in lung and K in olfactory bulb (Mn: F(1,6) = 6.28, *p* = 0.046; K: F(1,6) = 8.69, *p* = 0.026). In males, a marked and significant increase in lung Fe was observed (F(1,6) = 6.2, *p* = 0.047), while numerous changes were detected in frontal cortex, including significant increases in Mg, K and Zn (Mg: F(1,6) = 6.54, *p* = 0.043; K: F(1,6) = 6.88, *p* = 0.039; Zn: F(1,6) = 17.13, *p* = 0.006) and marginal increases in P and S (P: F(1,6) = 4.59, *p* = 0.076; S: F(1,6) = 5.08, *p* = 0.065). Thus, marked changes in metal concentrations in frontal cortex were seen in response to Fe only in females but after Fe + SO_2_ exposure in males and the corresponding changes were opposite in direction.

**Fig. 4:**
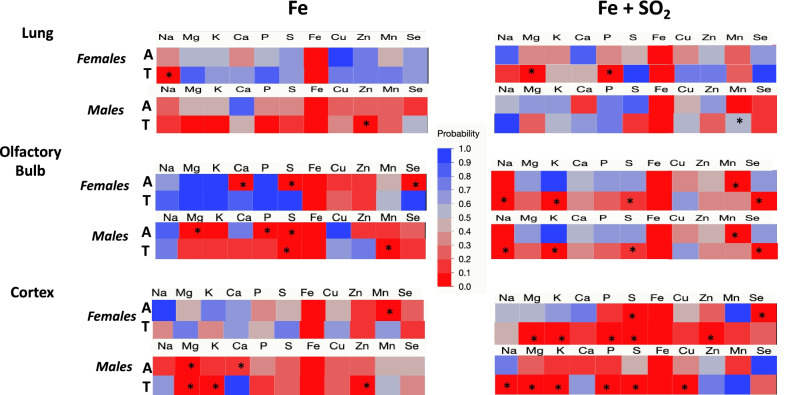
Changes in correlation p values of Fe with other trace elements and metals following exposures to Fe only (left column) or Fe + SO_2_ (right column) in lung, olfactory bulb and cortex (top, middle and bottom rows, respectively in females or males as indicated exposed to filtered air (A) or Fe or Fe + SO_2_ (treated, T). * = significant correlation with Fe; n = 4/group

To assess metal dyshomeostasis, correlations among metals within each region were examined. Figure 4 shows correlations of Fe with other metals in groups treated with Fe only (left column) or Fe + SO_2_ (right column) for females and males exposed to either filtered air (A) or treatment (T: Fe or Fe + SO_2_). In lung (Fig. 4 top row), Fe was not correlated with any other metal/element in air-exposed females. However, significant correlations emerged with Na post Fe only exposure, and with both Mg and P after Fe + SO_2_ exposure. Similarly, Fe was not correlated with other metals/elements in lung of air-treated males; this was not altered by Fe + SO_2_ treatment, while correlations with Zn emerged following Fe only.

In olfactory bulb (Fig. 4 middle row), Fe was significantly correlated with Ca, S and Se in air-treated females in the Fe only exposure group, with all of these correlations eliminated post Fe only exposure. In air-exposed males in the Fe only exposure group, correlations of Fe with Mg, P and S were found; Fe only exposure eliminated the correlations with Mg and P, while the correlation of Fe with S was retained, and a new correlation with Mn emerged. In contrast, following Fe + SO_2_ exposures, an increase in Fe correlations with other metals was observed in females, that included emergence of correlations of Fe with Na, K, S and Se, while the correlation of Fe with Mn that was observed in air-treated females disappeared. Similarly, in air-treated males, Fe correlated only with Mn, but following the Fe + SO_2_ exposure, new correlations of Fe emerged with Na, K, S and Se emerged, while the correlation with Mn disappeared.

The greatest number of increased correlations of Fe with other metals occurred following Fe + SO_2_ exposures in cortex. In frontal cortex of air-exposed females (Fig. 4 bottom row), Fe was only associated with Mn, and this correlation was not evident in Fe only exposed females. In frontal cortex of air-treated males, Fe only exposure was initially correlated with Mg and Ca, while post Fe only exposure, the correlation of Fe with Mg was retained, and new correlations emerged between Fe with K and Zn. In the Fe + SO_2_ exposure condition, Fe was initially associated with S and Se in the air-treated females, with Fe + SO_2_ exposure further increasing correlations to include Fe with Mg, K, P, S, and Zn. While no correlations were seen in air-exposed males in the Fe + SO_2_ exposure condition, here too the Fe + SO_2_ exposure resulted in new correlations of Fe with Na, Mg, K, P, S, and Cu.

**Fig. 5:**
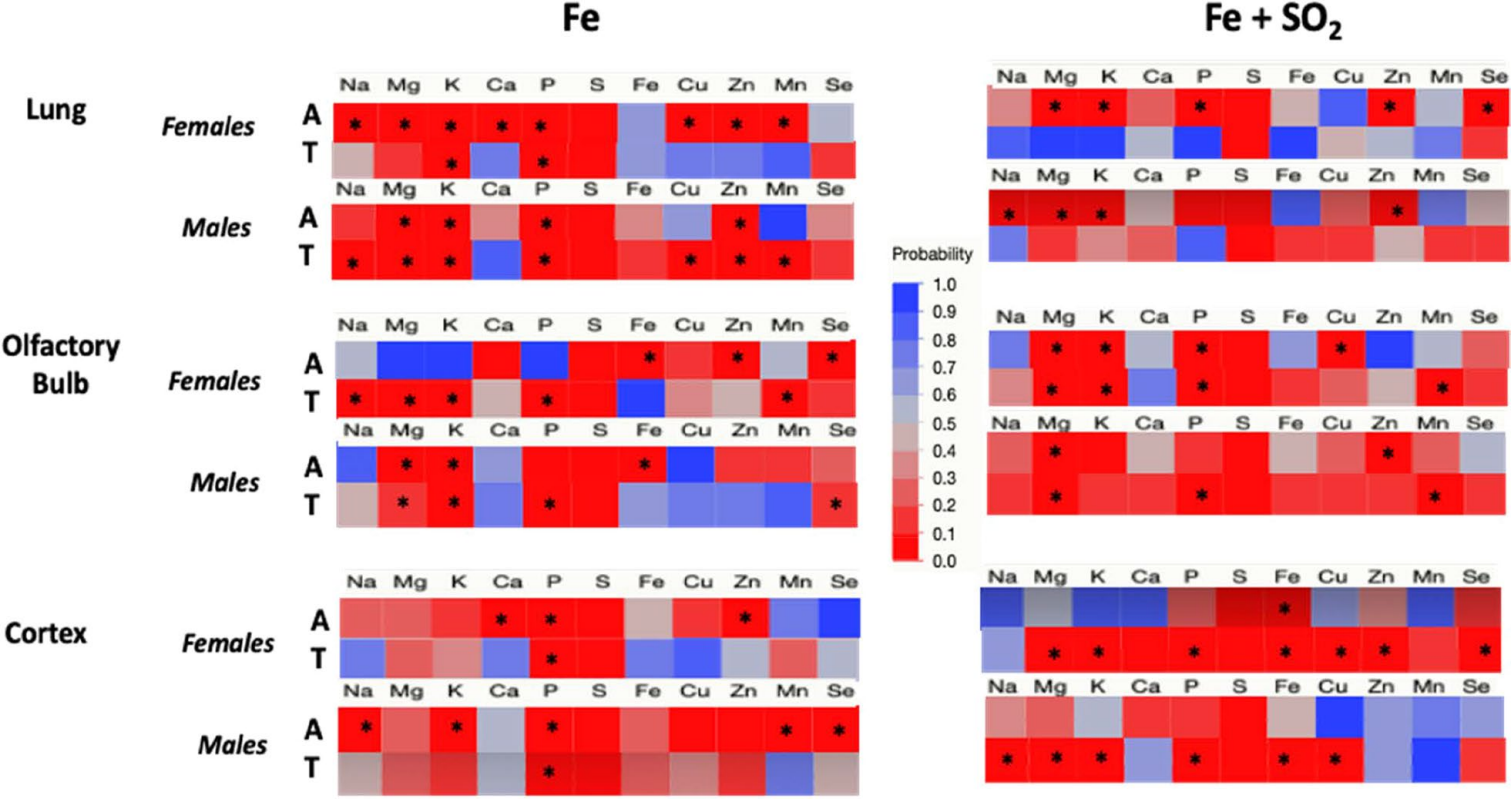
Changes in correlation p values of S with other trace elements and metals following exposures to Fe only (left column) or Fe + SO_2_ (right column) in lung, olfactory bulb and cortex (top, middle and bottom rows, respectively in females or males as indicated exposed to filtered air (A) or Fe or Fe + SO_2_ (treated, T). * = significant correlation with S; n = 4/group

Figure 5 depicts the same data but for correlations of S with other metals/elements. In lung (top row) of air-exposed females in the Fe only exposure condition, S was correlated with numerous other metals, including Na, Mg, K, Ca, P, Cu, Zn and Mn, and in the Fe + SO_2_ exposure condition, S was correlated with Mg, K, P, Zn and Se in air-exposed females. With the exception of the correlation of S with K and P in the Fe only exposed group, these correlations disappeared following both Fe and Fe + SO_2_ exposures. Air-treated males within the Fe only treatment group showed correlations of S with Mg, K, P and Zn, all of which were retained following Fe only exposure, and with new correlations of S with Na, Cu and Mn emerging. In contrast, in the Fe + SO_2_ condition, S correlated with Na, Mg, K and Zn in air-treated males, but all of these correlations disappeared following Fe + SO_2_ exposures.

Correlations of S with other metals/elements in olfactory bulb (middle row) of females were completely altered in the Fe only exposure condition, with initial correlations of S with Fe, Zn and Se in air-treated females, but altered to correlations of S with Na, Mg, K, P and Mn subsequent to Fe only exposure. Corresponding data in the Fe + SO_2_ group revealed initial correlations of S with Mg, K, P and Cu in the air-exposed females, with Cu disappearing and Mn added following Fe + SO_2_. In air-treated males within the Fe only exposed group, S was correlated with Mg, K and Fe, but correlations with Fe disappeared, while new correlations of S emerged with P and Se. In the Fe + SO_2_ condition, S initially correlated with Mg and Zn in air-treated males, with the Zn correlation disappearing and new correlations with P and Mn emerging post combined Fe and SO_2_ exposure.

Fe + SO_2_, but not Fe only exposures, markedly increased correlations of S in female frontal cortex (bottom row), with correlations changing from Fe only to include Mg, K, P, Fe, Cu, Zn and Se following Fe + SO_2_ exposure, whereas the correlations of S with Ca, P and Zn in air-treated females within the Fe only exposed group subsequently changed to a correlation only with P. In male frontal cortex, Fe only exposure eliminated correlations of S with Na, K, Mn and Se that were seen in air-treated males with only a correlation between S and P retained. In contrast, an absence of correlations of S with other elements/metals was observed in the air-exposed male frontal cortex of the Fe + SO_2_ group, with numerous correlations emerging post Fe + SO_2_ exposure, including correlations of S with Na, Mg, K, P, Fe and Cu. 

### Brain neurotransmitter analyses

**Fig. 6:**
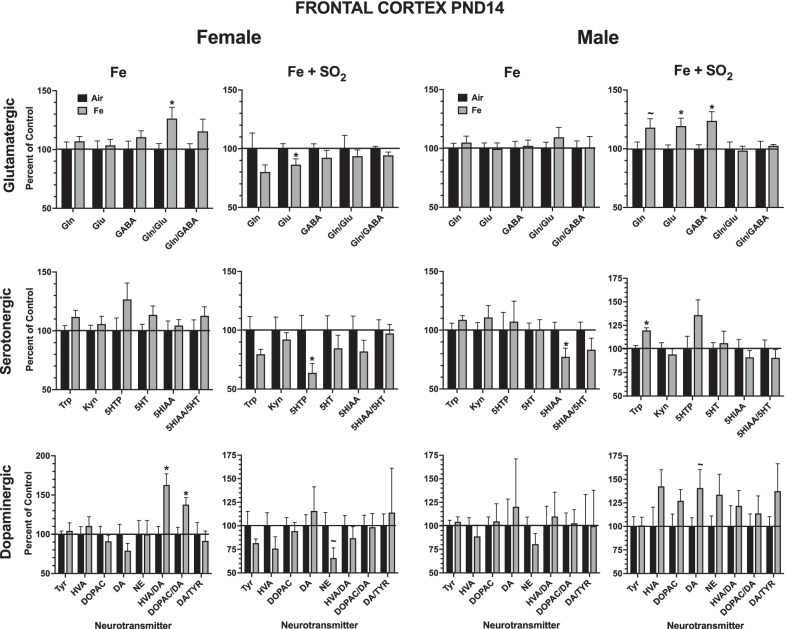
Group mean ± S.E. percent change relative to filtered air control of frontal cortex neurotransmitter levels at PND14 in males and females exposed to Fe only or Fe + SO_2_. * = significantly different from filtered air control; ~  = marginally different from filtered air control; n = 8/group. DA = dopamine; DOPAC = 3,4-dihydroxyphenylacetic acid; HVA = homovanillic acid, Tyr = tyrosine; NE = norepinephrine, Gln = glutamine; Glu = glutamate; GABA = gabba-aminobutyric acid; 5HT = serotonin; 5-HIAA = 5 hydroxyindoleacetic acid; 5-HTP = 5-hydroxytryptophan; Kyn = kynurenine; n = 7–8/group

Levels of brain neurotransmitters in frontal cortex at PND14 are shown in Fig. 6 as percent of filtered air control values. For females, changes in frontal cortex in response to Fe only exposure or Fe + SO_2_ exposures were limited, with the most marked effects being increases in dopamine turnover in response to Fe only, with significant increases in the ratios of both homovanillic (HVA) acid (F(1,14) = 12.82, *p* = 0.003) and of DOPAC to DA (F(1,14) = 8.75, *p* = 0.01). In males, the most notable effects were the significant increases in frontal cortical levels of glutamine (F1,14) = 3.55, *p* = 0.0806), glutamate (F(1,14) = 6.56, *p* = 0.023) and gamma aminobutyric acid (GABA; F(1,14) = 8.11, *p* = 0.013); trends consistent with increases in levels of dopaminergic neurotransmitters were found but were not significant.

**Fig. 7:**
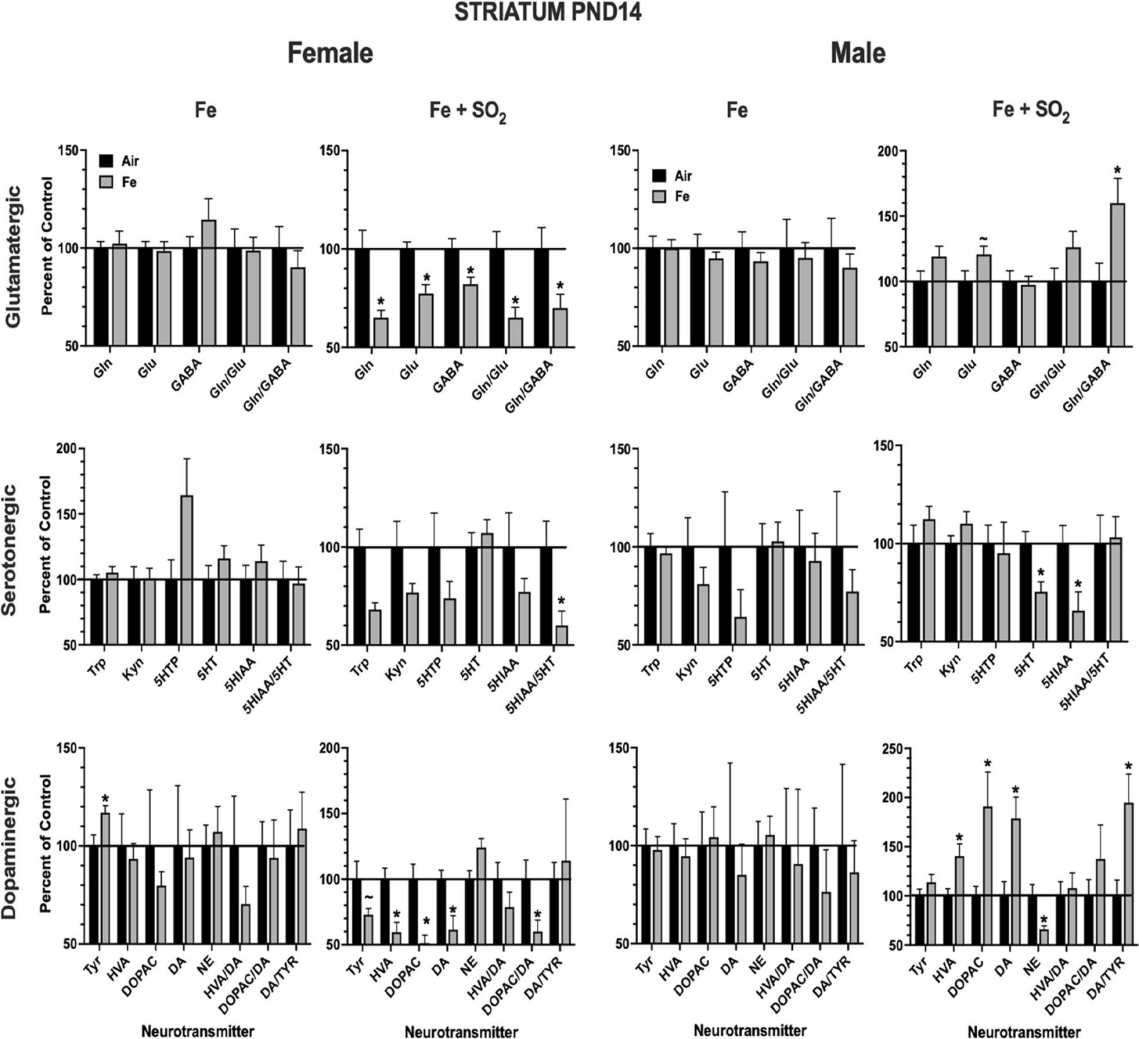
Group mean ± S.E. percent change relative to filtered air control of striatal neurotransmitter levels at PND14 in males and females exposed to Fe only or Fe + SO_2_. * = significantly different from filtered air control; ~  = marginally different from filtered air control; n = 8/group. DA = dopamine; DOPAC = 3,4-dihydroxyphenylacetic acid; HVA = homovanillic acid, Tyr = tyrosine; NE = norepinephrine, Gln = glutamine; Glu = glutamate; GABA = gabba-aminobutyric acid; 5HT = serotonin; 5-HIAA = 5 hydroxyindoleacetic acid; 5-HTP = 5-hydroxytryptophan; Kyn = kynurenine; n = 9–10/group

Corresponding changes in striatum are presented in Fig. 7 and show that Fe + SO_2_ exposure had far more impact in striatum in both sexes. For example, in females, marked reductions were seen in levels of glutamatergic neurotransmitters and turnover (glutamine (F(1,18) = 11.68, *p* = 0.003; glutamate (F(1,18) = 15.76, *p* = 0/0.0009; GABA (F(1,18) = 7.94, *p* = 0.011; glutamine/glutamate (F(1,18) = 11.53, *p* = 0.003; glutamate/GABA (F(1,18) = 5.39, *p* = 0.032), along with reductions in serotonin (5HT) turnover, i.e., 5 hydroxyindole acetic acid (5HIAA)/5HT (F(1,18) = 7.12, *p* = 0.0156), and in dopaminergic neurotransmitters and turnover (tyrosine, F(1,18) = 3.52, *p* = 0.077; HVA (F(1,18) = 12.94, *p* = 0.002; DOPAC (F(1,18) = 14.36, *p* = 0.001; DA (F(1,18) = 9.3, *p* = 0.007; DOPAC/DA (F(1,18) = 5.55, *p* = 0.03). In the case of males, Fe + SO_2_ exposure likewise reduced some serotonergic neurotransmitters, specifically 5HT F(1,17) = 9.49, *p* = 0.007) and 5 HIAA (F(1,17) = 6.64, *p* = 0.02), and, in contrast to females, markedly increased the dopaminergic neurotransmitters HVA (F(1,17) = 8.16, *p* = 0.01), DOPAC (F(1,17) = 6.95, *p* = 0.017) and DA (F(1,17) = 9.49, *p* = 0.007) and DA/tyrosine (F(1,17) = 8.56, *p* = 0.009), while reducing levels of norepinephrine (NE) (F(1,17) = 7.3, *p* = 0.015).

By PND60, few and predominately marginal changes in frontal cortex were seen in females (Additional file 1: Fig. 1), while males primarily showed marginal reductions in serotonin and its metabolite 5HIAA and turnover in response to Fe only exposures. Similarly, significant changes in striatal neurotransmitters were not apparent at PND60 (Additional file 1: Fig. 2), with the exception in females of increases in 5HT turnover after Fe only (F(1,14) = 5.16, *p* = 0.039) and of DA turnover (DOPACC/DA, F(1,13) = 7.01, *p* = 0.02) in response to Fe + SO_2_ exposure.

### Inflammation and oxidative stress

**Fig. 8:**
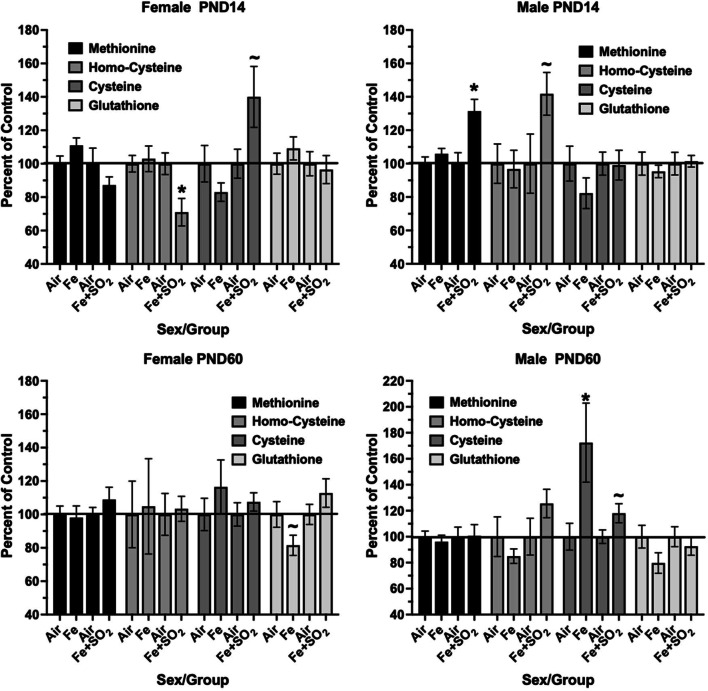
Group mean ± S.E. percent change relative to filtered air control in frontal cortex markers of trans-sulfuration at PND14 (top row) or PND60 (bottom row) in males and females exposed to Fe only or Fe + SO_2_ as indicated. * = significantly different from filtered air control; ~  = marginally different from filtered air control; n = 8/group. Met = methionine; H-Cys = homocysteine; Cys = cysteine; GSH = glutathione; n = 7–8/group

*Frontal Cortex Trans-Sulfuration Markers*—Several markers of the trans-sulfuration pathway were assessed in frontal cortex and are plotted as percent of control in Fig. 8. As it shows, significant changes in this pathway were seen only after Fe + SO_2_ at PND14. Specifically, these included significant reductions in homocysteine F(1,14) = 7.66, *p* = 0.015) and marginal increases in cysteine (F(1,14) = 3.91, *p* = 0.068) in females, and significant increases in methionine (F(1,14) = 10.67, *p* = 0.006) and marginal increases in homocysteine (F(1,14) = 3.66, *p* = 0.077) in males. The only significant change were increases in cysteine in males in response to Fe only at PND60.

**Fig. 9:**
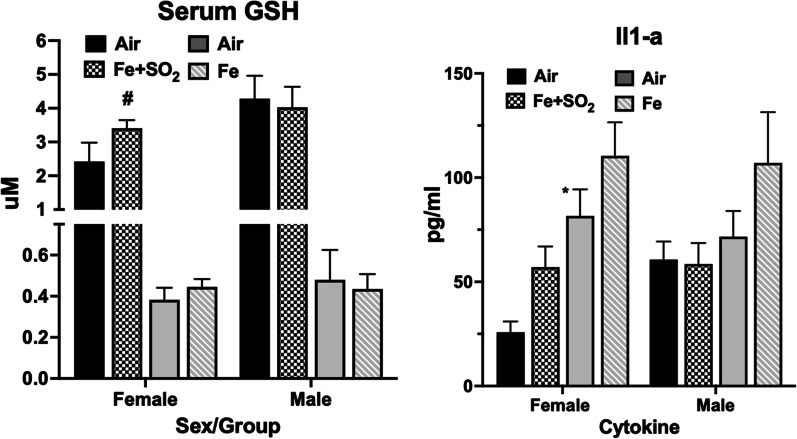
Group mean ± S.E. levels of serum glutathione (µM; left panel) and Il1-a (pg/ml; right panel) in males and females (as indicated) exposed to filtered air or to Fe (GSH: n = 10/group; ll-1a: n = 9–10/group) or Fe + SO_2_ (GSH: n = 6–8/group; Il1-a: n-3–8/group); * = significantly different from same sex filtered air control; ~  = marginally different from same sex filtered air control

*Serum Glutathione (GSH)—*Serum levels of glutathione were selectively increased in females in response to Fe + SO_2_ exposure (Fig. 9, left panel) by approximately 38%, an effect that just missed statistical significance (F(1,22) = 4.01, *p* = 0.0576). No effects were seen in response to Fe + SO_2_ exposure in males, nor did Fe only alter serum GSH in either sex.

*Cytokines*—Levels of IL-1a in serum were the only detectable cytokines at PND14 and are shown in Fig. 9, right panel. As it shows, increases in females in mean levels of IL-1a (F(1,10) = 6.06, *p* = 0.034) were found after Fe + SO_2_ exposure, with similar but non-significant trends in response to Fe only in males. Exposures to Fe + SO_2_ did not produce changes in IL-1a in males.

## Discussion

This study sought to determine whether Fe as a contaminant of air pollution exposures would reproduce features previously seen in response to postnatal and/or gestational ambient ultrafine particle exposures of mice. Brain Fe overload can result in oxidative stress and ferroptosis [79, 80], and the brain has no apparent mechanisms to excrete Fe to the periphery [81, 82]. Two general findings emerged from this study. First, comparisons of the number and magnitude of effects indicate a greater overall toxicity of Fe + SO_2_ as compared to Fe only exposures (Table 1) in both sexes, as shown by a broader set of changes in correlations of Fe + SO_2_ with other metals and trace elements, and in neurotransmitter changes at postnatal day 14, as well as female specific changes in serum GSH and the inflammatory cytokine, IL-1a. Such findings suggest that the chemical mixture clearly matters. The greater effects of Fe + SO_2_ may be consistent with reports that sulfide can produce acidic aerosols that can dissolve transition metals [83, 84], and other studies reporting that SO_2_ increased uptake of Fe_2_O_3_ in mouse bronchial epithelium and altered its intracellular distribution [66].

**Table 1:** Summary of Changes in Response to Fe Only or to Combined Fe + SO_2_

	Females		Males	
	Fe	Fe + SO_2_	Fe	Fe + SO_2_
Body weight		↓		
Lung metal	↓P	↓Mn	↑Ca, Na	↑Fe
Olfactory Bulb matals	↑Zn	↓K		
Frontal cortex metals	↓Mg, K, Ca, P, S, Cu, Zn			↑Mg, K, Zn
Lung Fe correlations	Na	Mg, P	Zn	
Olfactory bulb Fe correlations	Ca, S, Se	Na, K, S, Mn, SE	Mg, P, Mn	Na, K, S, Mn, Se
Frontal cortex Fe correlations	Mn	Mg, K, P, Zn, Se	K, Ca, Zn	Na, Mg, K, P, S, Cu
Lung Fe correlations	Na, Mg, Ca, Cu, Mn	Mg, K, P, Zn, Se	Na, Cu, Mn	Na, Mg, K, Zn
Olfactory bulb S correlations	Na, Mg, K, P, Fe, Zn, Mn, Se	Cu, Mn	P, Fe, Se	P, Zn, Mn
Frontal cortex S correlations	Ca, Zn	↓Mg, K, P, Cu, Zn, Se	Na, K, Mn, Se	Na, Mg, K, P, Fe, Cu
Frontal cortex Glutamatergic PND14	↑Gln/Glu	↓Glu		~ ↑Gln, ↑Glu, GABA
Frontal cortex Serotonergic PND14		↓5HTP	↓5HIAA	↑Trp
Frontal cortex Dopaminergic PND14	↑HVA/DA, DOPAC/DA	~ ↓NE		~ ↑DA
Striatal Glutamatergic PND14		↓Gln, Glu, GABA, Gln/Glu, Glu/GABA		~ ↑Glu, ↑Glu/ GABA
Striatal Serotonergic PND14		↓SHIAA/SHT		↓5HIAA, 5HT
Striatal Dopaminergic PND14	↑Tyr	~ ↓Tyr, ↓HVA, DOPAC, DA, D		↑HVA, DOPADA, DA/Tyr, ↓NE
Frontal Cortex Trans-Sulfuration PND14		↓H-Cys, ~ ↑Cys	~ ↑Cys	↑Met, ~ ↑H-Cys
Serum Glutathione		~ ↑		
Serum IL1a		↑		
Lateral Ventricle area	↑	↓		
Lateral Ventricle perimeter	↑	↑		

Summary of changes in response to Fe only or combined Fe + SO_2_. ↑, significant increase; ↓, significant decrease; ~  , marginally significant; DA, Dopamine; DOPAC, 3,4-dihydroxyphenylacetic acid; HVA, Homovanillic acid; Tyr, Tyrosine; NE, Norepinephrine; Gln, Glutamine; Glu, Glutamate; GABA, Gabba-aminobutyric acid; 5HT, Serotonin; 5-HIAA, 5 hydroxyindoleacetic acid; 5-HTP, 5-hydroxytryptophan; Kyn, Kynurenine; Met, Methionine; H-Cys, Homocysteine; Cys, Cysteine; GSH, Glutathione

Another observation is an apparent greater vulnerability of females than males to Fe and Fe + SO_2_ exposures, based on the selective effects in females in body weight, GSH and IL-1a, and in frontal cortex metal level changes following Fe only. Sex differences in brain Fe levels and differences in Fe homeostasis have been reported in humans and in animal models. For example, human females have been reported to have lower levels of the Fe export protein ferritin in brain than do males [85], while another study reported lower total subcortical brain Fe in women from midlife relative to men and to younger women [86]. Interestingly, differences in Fe status are seen even during infancy [87]. Nevertheless, an understanding of sex differences in early Fe handling and function and its impact on brain development is lacking.

Metal dyshomeostasis was found after both Fe only and Fe + SO_2_ exposures in both sexes as evidenced not only by altered levels of metals in lung and brain, but additionally as seen in alterations in correlations of both Fe and S with other metals/elements within these regions, which was particularly striking in frontal cortex. Metal dyshomeostasis has been implicated in neurodevelopmental disorders [52–55] as well as in neurodegenerative diseases and disorders [88, 89]. Here too, both sex and chemical mixture contributed to differences in outcome. For example, in frontal cortex, Fe only exposure reduced levels of multiple metals/trace elements in females, with no such effects observed in response to Fe + SO_2_; in contrast, males showed increases in several metals/elements in frontal cortex following Fe + SO_2_ exposure, whereas Fe alone had no effect. Further, increases in levels of Fe were marginal in female lung after Fe only exposure, but significant in males after Fe + SO_2_ exposures.

Multiple neurochemical changes were seen in response to ambient UFP exposures in both frontal cortex and striatum of mice in our prior studies of exposures to concentrated ambient UFPs [68]. Similarly, neurochemical changes were observed in the current study. These included changes in dopaminergic function, particularly in striatum in response to Fe + SO_2_ exposures, again with effects that were of opposite direction by sex, with reductions seen in females, whereas increases occurred in males. Similarly, our prior studies of ambient UFPs have revealed evidence of sex specific changes in dopaminergic function, including alterations at postnatal days 14 and 55 [14] and at PND270 [68]. Sex differences in DA function and corresponding consequences have long been known, including in human studies [90]. Reports in rats include enhanced DA release in female rats [91], and of maximal velocity of DA reuptake in females [92] as compared to males. One suggested basis for such differences has been a greater DA terminal density in females compared to males in caudate nucleus [92]. Males were reported to show a greater overproduction of striatal D1 and D2 receptors in comparison to females [93]. A potential role for Fe contamination of UFPs in DA changes is highly plausible, as DA metabolism can produce neurotoxic species, particularly quinones which can then form neurotoxic intermediates via Fe-dependent reactions [94]. DA can also increase uptake of labile Fe by macrophages resulting in oxidative stress [95]. In addition, DA quinones can react with S-containing compounds such as l-cysteine and reduced glutathione tripeptide, both of which are present in high concentrations in brains, ultimately leading to toxic produces such as 5-S-Cys-dopamine that can lead to DA neuronal death [96].

Of particular note in the current study, increases in frontal cortex glutamine, glutamate and GABA and of striatal glutamate and glutamate/GABA were seen in males in response to Fe + SO_2_. Such findings are consistent with those produced by developmental exposures to ambient UFPs in our prior studies of increases in males observed at both PND14 and PND55 [14] following UFP exposures with a mean of 96 ug/m^3^. Such findings are reminiscent of excitatory/inhibitory imbalance, considered a key feature of autism and schizophrenia [97]. Again, however, consistent with sex differences in response to Fe, Fe + SO_2_ decreased glutamate in females, whereas increases in glutamate turnover occurred in response to Fe only in females. Sex- and brain-region related differences in levels of glutamate, GABA and aspartate have previously been documented, and include differences seen in the early postnatal period in rat brain [98]. In juvenile rats, for example, GLU signaling molecules were found at significantly lower levels in females [99]. Sex differences in GLU function are likewise reported in humans, and have included more rapid age-related declines in glutamate in males [100]. Collectively, such findings suggest a role for Fe in the persistence of UFP-induced excitatory/inhibitory imbalance we previously observed seen in males; importantly this is considered a key feature of autism and schizophrenia [97], both of which are male-biased disorders.

Mechanistically, glutamate has the potential for neurotoxic interactions with Fe, specifically in the framework of ferroptosis, wherein glutamate can inhibit uptake of cystine by the cystine/glutamate antiporter resulting in glutathione depletion, thereby facilitating Fe-produced oxidative damage [101, 102]. The glutamine-glutamate cycle is within the brain’s trans-sulfuration pathway [103, 104]. Notably, a recent study suggested a stronger regulatory control between peripheral Fe and glutamate metabolism in females [105].

**Fig. 10:**
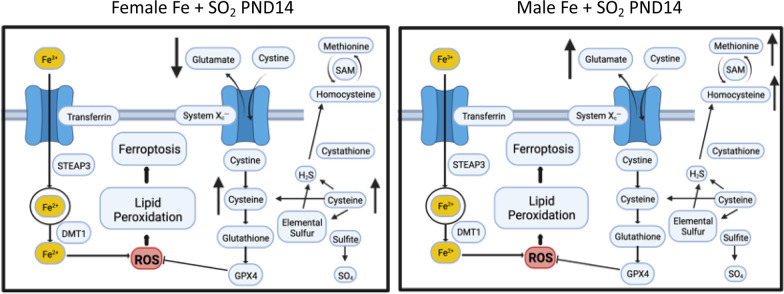
Schematic linking the brain trans-sulfuration pathway, glutamate, lipid peroxidation and ferroptosis with changes in trans-sulfuration markers and glutamate, that differed in females (decreased glutamate, increased cysteine) vs males (increased methionine and homocysteine as well as glutamate) at postnatal day 14 and suggest different mechanisms may be operative in the two sexes**.** Created with BioRender.com

The trans-sulfuration pathway is also well known to interact with glutamatergic systems and functions [106–108] and thus it is possible that elevations in frontal cortex glutamate seen in males, and reductions in glutamate seen in females in response to Fe + SO_2_ could be due to oxidative stress-related effects of Fe. However, changes in trans-sulfuration systems appear to occur in a sex-dependent capacity and can be influenced by hormones [109–112]. Such sex differences were reflected in the profiles of both trans-sulfuration markers and glutamatergic changes seen in response to Fe + SO_2_ in this study (Fig. 10). Correspondingly, these findings suggest that mechanistic links between these systems, and thus ultimately, mechanistic links both in excitotoxicity and redox status, and their direction of causation, will likely differ by sex.

Interestingly, although our previous research on postnatal exposure conducted in Rochester, NY shows strong male-biased early toxicity to concentrated ambient ultrafine particles, our previous work on gestational exposures conducted in Sterling, NY shows significant female and male effects. These gestational exposures, however, as carried out in Sterling NY were more enriched with Fe as measured by Elemental X-Ray Fluorescence, with Fe levels indicating a 376 fold increase over filtered air (FA). Average Fe concentrations on filters from filtered air chambers were 3.8 ng/m^3^, while exposure filter values were 1,430 ng/m^3^ [18, 19, 113]. Future research is needed to explore the sex-dependent effect of Fe exposure in early gestation as compared to postnatal exposures.

Given that the ambient exposures include numerous metals and organics as a mixture, and thus exposures to Fe only would not be expected to fully or explicitly reproduce all of the neurotoxic effects of ambient air pollution, the current evidence suggests that Fe contamination of UFP does contribute to features of the neurotoxicity of ambient air pollution, with different impacts by sex and outcome measures. It should also be noted that the exposures used here produced a consistently high number concentration exposure of pure Fe particles, exceeding what would be seen in Fe ambient environmental exposure levels by number. Nevertheless, an increased understanding of the role of metal contaminants in air pollution as a contributor to the etiology of neurodevelopmental disorders and neurodegenerative diseases is critical given that these are life-long exposures beginning in utero. Such an understanding can guide the development of more realistic focused animal exposure models of human AP exposure as well as allow refinement of epidemiological studies, and, consequently, a more meaningful approach to mechanistic studies. In addition, it would be potentially informative in population studies, for example, using geographic mapping of exposures to various metal contaminants of AP, along with gases such as SO_2_, where available, in relation to diagnostic or incidence data for various neurodevelopmental disorders and neurodegenerative diseases [30]. Such studies would also point to potential intervention strategies for neurodevelopmental disorders and neurodegenerative diseases. Understanding the components of AP relating to neurotoxicity is of direct translational relevance to public health protection, as it can provide information pertinent to regulations of exposures, including the need for new or more stringent regulations.

## Supplementary Information

**Additional file 1. Supplemental Figure 1:** Group mean ± S.E. percent change relative to filtered air control of frontal cortex neurotransmitter levels at PND60 in males and females exposed to Fe only or Fe + SO_2_. *= significantly different from filtered air control; ~=marginally different from filtered air control; n=8/group. DA=dopamine; DOPAC=3,4-dihydroxyphenylacetic acid; HVA=homovanillic acid, Tyr=tyrosine; NE=norepinephrine, Gln=glutamine; Glu=glutamate; GABA=gabba-aminobutyric acid; 5HT=serotonin; 5-HIAA=5 hydroxyindoleacetic acid; 5-HTP=5-hydroxytryptophan; Kyn=kynurenine; n=7-8/group. **Supplemental Figure 2:** Group mean ± S.E. percent change relative to filtered air control of striatal neurotransmitter levels at PND60 in males and females exposed to Fe only or Fe + SO_2_. *= significantly different from filtered air control; ~=marginally different from filtered air control; n=8/group. DA=dopamine; DOPAC=3,4-dihydroxyphenylacetic acid; HVA=homovanillic acid, Tyr=tyrosine; NE=norepinephrine, Gln=glutamine; Glu=glutamate; GABA=gabba-aminobutyric acid; 5HT=serotonin; 5-HIAA=5 hydroxyindoleacetic acid; 5-HTP=5-hydroxytryptophan; Kyn=kynurenine; n=9-10/group.

## Data Availability

The datasets used and/or analyzed in the current study are available from the corresponding author on reasonable request.
