# Supplementary material for: The potential involvement of inhaled iron (Fe) in the neurotoxic effects of ultrafine particulate matter air pollution exposure on brain development in mice
Source: Part Fibre Toxicol. 2022 Aug 9;19:56. doi: 10.1186/s12989-022-00496-5 (PMC9364598; doi:10.1186/s12989-022-00496-5)
Supplement: Supplementary file 1 — Additional file 1. Supplemental Figure 1: Group mean ± S.E. percent change relative to filtered air control of frontal cortex neurotransmitter levels at PND60 in males and females exposed to Fe only or Fe + SO2. *= significantly different from filtered air control; ~=marginally different from filtered air control; n=8/group. DA=dopamine; DOPAC=3,4-dihydroxyphenylacetic acid; HVA=homovanillic acid, Tyr=tyrosine; NE=norepinephrine, Gln=glutamine; Glu=glutamate; GABA=gabba-aminobutyric acid; 5HT=serotonin; 5-HIAA=5 hydroxyindoleacetic acid; 5-HTP=5-hydroxytryptophan; Kyn=kynurenine; n=7-8/group. Supplemental Figure 2: Group mean ± S.E. percent change relative to filtered air control of striatal neurotransmitter levels at PND60 in males and females exposed to Fe only or Fe + SO2. *= significantly different from filtered air control; ~=marginally different from filtered air control; n=8/group. DA=dopamine; DOPAC=3,4-dihydroxyphenylacetic acid; HVA=homovanillic acid, Tyr=tyrosine; NE=norepinephrine, Gln=glutamine; Glu=glutamate; GABA=gabba-aminobutyric acid; 5HT=serotonin; 5-HIAA=5 hydroxyindoleacetic acid; 5-HTP=5-hydroxytryptophan; Kyn=kynurenine; n=9-10/group. [file 12989_2022_496_MOESM1_ESM.docx]

**Supplemental Materials**

**
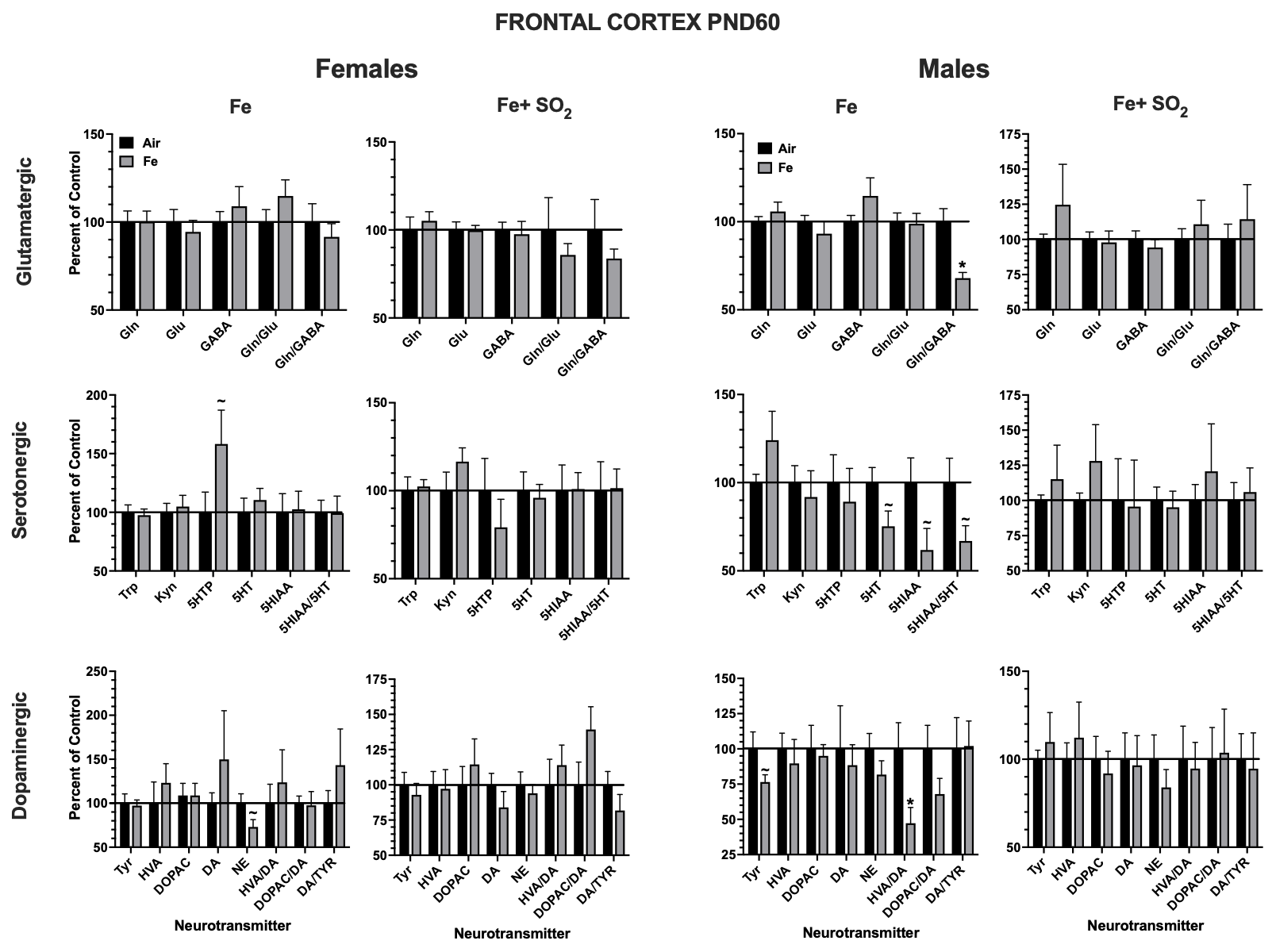
**

**Supplemental Figure 1:** Group mean ± S.E. percent change relative to filtered air control of frontal cortex neurotransmitter levels at PND60 in males and females exposed to Fe only or Fe + SO_2_ . *= significantly different from filtered air control; ~=marginally different from filtered air control; n=8/group. DA=dopamine; DOPAC=3,4-dihydroxyphenylacetic acid; HVA=homovanillic acid, Tyr=tyrosine; NE=norepinephrine, Gln=glutamine; Glu=glutamate; GABA=gabba-aminobutyric acid; 5HT=serotonin; 5-HIAA=5 hydroxyindoleacetic acid; 5-HTP=5-hydroxytryptophan; Kyn=kynurenine; n=7-8/group.

**
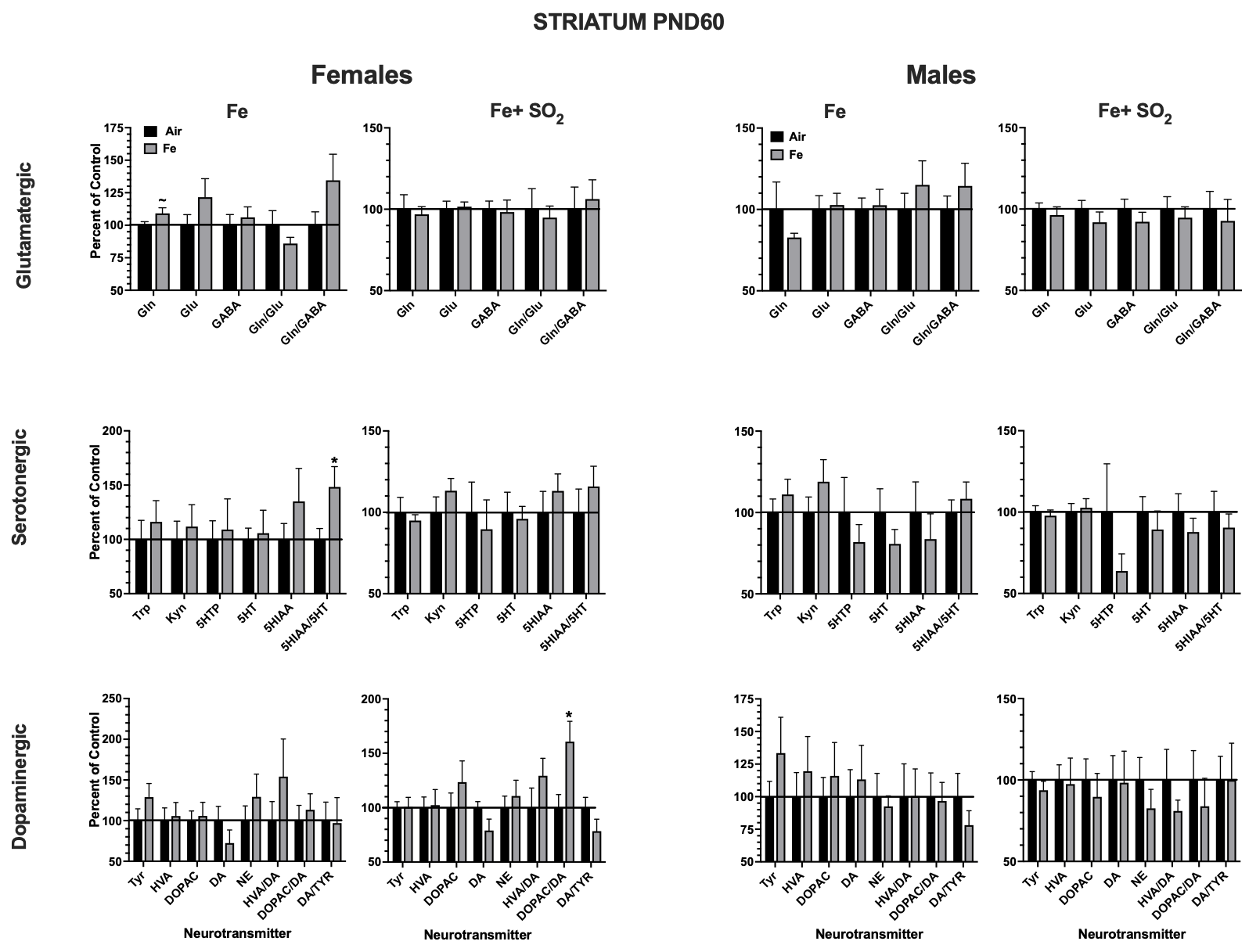
**

**Supplemental Figure 2:** Group mean ± S.E. percent change relative to filtered air control of striatal neurotransmitter levels at PND60 in males and females exposed to Fe only or Fe + SO_2_. *= significantly different from filtered air control; ~=marginally different from filtered air control; n=8/group. DA=dopamine; DOPAC=3,4-dihydroxyphenylacetic acid; HVA=homovanillic acid, Tyr=tyrosine; NE=norepinephrine, Gln=glutamine; Glu=glutamate; GABA=gabba-aminobutyric acid; 5HT=serotonin; 5-HIAA=5 hydroxyindoleacetic acid; 5-HTP=5-hydroxytryptophan; Kyn=kynurenine; n=9-10/group.

**Supplemental Table 1: Quality Control Outcomes for Metals Analyses**

| QC type | Identifer | Proficiency sample ID | Units | Na | Mg | K | Ca | P | S | Fe | Cu | Zn | Mn | Se |
| --- | --- | --- | --- | --- | --- | --- | --- | --- | --- | --- | --- | --- | --- | --- |
| Calibration Verification | ICV |  |  | 102% | 103% | 103% | 103% | 102% | 110% | 104% | 111% | 105% | 107% | 101% |
| Calibration Verification | ICV |  |  | 104% | 106% | 104% | 105% | 103% | 107% | 104% | 105% | 101% | 108% | 101% |
| Calibration Verification | ICV |  |  | 102% | 103% | 102% | 101% | 101% | 110% | 103% | 109% | 104% | 105% | 97% |
| Calibration Verification | ICV |  |  | 106% | 106% | 106% | 104% | 103% | 110% | 107% | 112% | 108% | 108% | 99% |
| Calibration Verification | ICV |  |  | 105% | 105% | 104% | 103% | 103% | 110% | 105% | 111% | 106% | 107% | 99% |
| Calibration Verification | ICV |  |  | 102% | 103% | 101% | 101% | 101% | 109% | 101% | 108% | 103% | 106% | 98% |
| Calibration Verification | ICV |  |  | 104% | 104% | 104% | 103% | 100% | 110% | 102% | 106% | 104% | 106% | 96% |
| Calibration Verification | ICV |  |  | 101% | 103% | 102% | 100% | 101% | 109% | 105% | 108% | 102% | 103% | 97% |
| Calibration Verification | ICV |  |  | 104% | 105% | 104% | 103% | 104% | 111% | 105% | 108% | 105% | 105% | 99% |
| Calibration Verification | ICV |  |  | 104% | 104% | 104% | 103% | 103% | 108% | 106% | 109% | 104% | 104% | 100% |
| Calibration Verification | ICV |  |  | 105% | 106% | 106% | 104% | 104% | 111% | 108% | 110% | 105% | 104% | 97% |
| Calibration Verification | ICV |  |  | 108% | 108% | 108% | 105% | 102% | 108% | 108% | 110% | 104% | 104% | 95% |
| Calibration Verification | ICV |  |  | 104% | 105% | 106% | 104% | 101% | 108% | 105% | 109% | 105% | 104% | 97% |
| Calibration Verification | ICV |  |  | 103% | 103% | 104% | 104% | 103% | 108% | 103% | 107% | 103% | 106% | 100% |
| Calibration Verification | ICV |  |  | 108% | 107% | 106% | 107% | 102% | 108% | 110% | 111% | 107% | 106% | 100% |
| Calibration Verification | ICV |  |  | 105% | 105% | 104% | 104% | 102% | 106% | 108% | 111% | 106% | 104% | 98% |
| Calibration Verification | ICV |  |  | 108% | 108% | 105% | 107% | 98% | 105% | 109% | 110% | 105% | 106% | 95% |
| Laboratory Control Solution | USGS PT | P76 |  | 105% | 97% | 95% | 81% | 107% | 101% |  |  |  |  |  |
| Laboratory Control Solution | USGS PT | P76 |  | 106% | 98% | 96% | 83% | 109% | 104% |  |  |  |  |  |
| Laboratory Control Solution | USGS PT | P76 |  | 108% | 100% | 99% | 84% | 107% | 105% |  |  |  |  |  |
| Laboratory Control Solution | USGS PT | P76 |  | 104% | 96% | 96% | 81% | 109% | 102% |  |  |  |  |  |
| Laboratory Control Solution | USGS PT | T-245 |  | 103% | 104% | 105% | 114% |  |  | 98% | 99% | 99% | 103% | 105% |
| Laboratory Control Solution | USGS PT | T-245 |  | 104% | 104% | 106% | 114% |  |  | 98% | 101% | 101% | 103% | 97% |
| Laboratory Control Solution | USGS PT | T-245 |  | 103% | 102% | 102% | 111% |  |  | 95% | 97% | 97% | 101% | 90% |
| Laboratory Control Solution | USGS PT | T-245 |  | 104% | 103% | 104% | 114% |  |  | 98% | 98% | 98% | 101% | 99% |
| Standard Reference Material | NIST 2976 |  |  | 99% | 93% | 103% | 88% | 88% | 89% | 96% | 106% | 101% | 98% | 92% |
| Standard Reference Material | NIST 2976 |  |  | 101% | 96% | 109% | 98% | 91% | 95% | 68% | 112% | 83% | 71% | 92% |
| Standard Reference Material | NIST 2976 |  |  | 103% | 96% | 107% | 97% | 90% | 93% | 87% | 113% | 86% | 88% | 96% |
| Standard Reference Material | NIST 2976 |  |  | 103% | 98% | 108% | 95% | 90% | 92% | 87% | 116% | 113% | 89% | 94% |
| Standard Reference Material | NIST 2976 |  |  | 100% | 94% | 105% | 94% | 87% | 90% | 110% | 112% | 84% | 111% | 92% |
| Standard Reference Material | NIST 2976 |  |  | 100% | 94% | 104% | 99% | 88% | 90% | 90% | 110% | 88% | 91% | 92% |
| Analysis dup | A dup |  |  | 10% | 5% | 6% | 5% | 6% | 9% | 1% | 0% | 0% | 0% | 0% |
| Analysis dup | A dup |  |  | 2% | 1% | 0% | 1% | 6% | 6% | 3% | 2% | 2% | 1% | 4% |
| Analysis dup | A dup |  |  | 5% | 6% | 4% | 7% | 0% | 4% | 3% | 2% | 2% | 3% | 25% |
| Analysis dup | A dup |  |  | 4% | 2% | 1% | 4% | 4% | 7% | 2% | 0% | 2% | 2% | 10% |
| Analysis dup | A dup |  |  | 3% | 3% | 2% | 0% | 5% | 5% | 4% | 0% | 1% | 2% | 14% |
| Analysis spike | A spk |  |  | 114% | 114% | 103% | 193% | 94% | 94% | 108% | 110% | 117% | 110% | 101% |
| Analysis spike | A spk |  |  | 122% | 106% | 94% | 177% | 54% | 63% | 102% | 102% | 105% | 10% | 94% |
| Analysis spike | A spk |  |  | 115% | 113% | 104% | 187% | 87% | 99% | 106% | 106% | 110% | 11% | 96% |
| Analysis spike | A spk |  |  | 127% | 107% | 107% | 187% | 67% | 71% | 104% | 105% | 106% | 10% | 96% |
|  |  |  |  |  |  |  |  |  |  |  |  |  |  |  |
